# Oral health status among Iranian veterans exposed to sulfur mustard: A case-control study

**DOI:** 10.4317/jced.52112

**Published:** 2015-04-01

**Authors:** Yunes Panahi, Taghi Azizi, Mohammad-Reza-Sadeghi Moghadam, Golshah Amin, Shahram Parvin, Amirhossein Sahebkar

**Affiliations:** 1 Chemical Injuries Research Center, Baqiyatallah University of Medical Sciences, Tehran, Iran; 2 Oral and Maxillofacial Pathology, Baqiyatallah University of Medical Sciences, Tehran, Iran; 3 Oral and Maxillofacial Pathology, Baqiyatallah University of Medical Sciences, Tehran, Iran; 4 Biotechnology Research Center, Mashhad University of Medical Sciences, Mashhad, Iran; 5 Metabolic Research Centre, Royal Perth Hospital, School of Medicine and Pharmacology, University of Western Australia, Perth, Australia

## Abstract

**Background:**

Sulfur mustard (SM) is a chemical warfare agent that has been repeatedly used since World War I. SM has chronic and deleterious effects on different body organs such as lungs, skin and eyes.

**Objectives:**

To determine dental and oral health status of chemical victims of SM who were exposed to SM during the Iraqi-Iran war.

**Material and Methods:**

In this case-control study, 100 male subjects exposed to SM were chosen as cases, and 100 non-exposed volunteers were chosen as controls. These groups were selected randomly according to their referral number, and were matched regarding age. Collection of information was performed using Oral Health Assessment Form designed by the World Health Organization. Quantitative and qualitative data were compared between the groups using independent samples t-test and Chi-square test, respectively.

**Results:**

There was a significant difference between the case and control groups with respect to the frequencies of oral candidiasis, pharyngeal erythema and/or hyperplasia, hairy tongue and reflux disease, being higher in the former group. There was also a positive association between the frequency of candidiasis and the percentage of disability; pharyngeal erythema and/or hyperplasia and use of salmeterol spray; and between hairy tongue and antibiotic use in the case group.

**Conclusions:**

Exposure to SM and the use of drugs for controlling long-term complications does not increase the risk of tooth decay, tooth loss, and intra and/or extra oral lesions in patients, but may be associated with increased incidence of oral candidiasis, pharyngeal erythema and/or hyperplasia, hairy tongue and reflux disease.samples of oral precancerous and cancerous lesions to test sensitivity and specificity and thus validate the clinical applicability of fluorescence imaging in (pre)cancerous diagnostics.

** Key words:**Sulfur mustard, oral health, candidiasis.

## Introduction

Sulfur mustard (SM) gas is a chemical warfare agent that has been used repeatedly since the First World War. This gas is produced easily, even by developing countries, and its military use has led to great casualties (1). SM was employed extensively by the Iraqi army against Iranian soldiers, and also Iranian and Iraqi civilians between 1983 and 1988, resulting in over 100,000 chemical casualties (2). Currently, a considerable number (~45,000) of SM-intoxicated subjects with chronic cutaneous, ocular and respiratory symptoms are living in Iran. SM is a yellow oily liquid with a stench of mustard or garlic in room temperature, and evaporates rapidly in hot and dry environments. SM affects different body organs and tissues by alkylation of nucleic acids and proteins (3). Its effects are long-lasting but not fatal (3% mortality in the second World-War) (1). SM is a strong blistering agent; each 0.1 mL is 20,000 times the required dose to cause blisters. There may be no signs or symptoms in the first 12 to 24 hours following exposure. In poisoning cases, erythema is usually seen prior to blistering (1). In many cases with SM-induced respiratory lesions, primary nasal and respiratory tract mucosal burn bronchitis develops within 4-6 weeks. In severe cases of exposure, necrosis of respiratory epithelium or oral mucosa results in the development of pseudomembrane and bronchopneumonia (4). Systemic absorption results in nausea, vomiting, hypotension, bradycardia and leukopenia following primary leukocytosis. Death is a result of infection, though it is uncommon (4,5).

SM has two types of effects on body: short-term and long-term. Its long term effects are due to its effects on intracellular components particularly DNA and proteins resulting in several biochemical imbalances and clinical manifestations (1-4) that are may not be completely controlled by available treatments, and necessitate new modalities (5-13). These effects cause mutagenic and carcinogenic changes in long-term and can affect the integrity of oral mucosa as well as the hematologic parameters (6-8). Hematologic complications might be the result of bone marrow suppression and subsequent decrease in the number of bone marrow precursor cells, causing aplastic anemia (9-11). SM can also promote tumorigenesis and increase the risk of pulmonary, bladder, pharyngeal, and gastric cancers (11-13), as well as leukemia and malignant melanoma (1). Chronic effects of SM also include persistent inflammation of the oral cavity, pharynx and larynx, inflammation and ulceration of the palate, nasopharynx, oropharynx and cancer of the larynx, with temporary aphonia (1). Moreover, oral mucosal lesions are likely to be seen in SM-intoxicated patients either as a result of long-term effects of SM on immunological parameters, or the use of drugs for treatment of exposure complications (10). The aim of the present study was to determine dental and oral health status of chemically injured subjects exposed to SM compared with those of healthy subjects.

## Material and Methods

This case-control study was performed on 200 subjects who referred to the Dental Clinic of the Baqiyatallah Hospital, Tehran, Iran. Patients were selected for this study randomly according to their referral number. Classification of subjects as case or control was based on the exposure to SM, and 100 subjects were included in each group. Percentage of disability for each subject in the case group was obtained from medical documents certified by the Veteran’s Affair of Iran (Janbazan Foundation). The procedure of determining disability percentage in chemically-injured subjects is based on the presence and severity (classified as mild, moderate or severe) of chronic cutaneous symptoms, delayed keratitis, and bronchiolitis (according to the findings of high-resolution computed tomography). Data collection was performed through observation and interview using WHO Oral Health Assessment Form 1997 (14) plus added questions regarding the percentage of disability, smoking habit, presence of other chronic complications due to SM exposure, location of exposure, and educational status. WHO Oral Health Assessment Form was used in the present study due to lacking any validated and standardized questionnaire for the evaluation of oral and dental health status in Iran. All patients were examined by an expert dentist. Radiographic pictures and digital photos of suspected lesions were taken if needed. Mouth dryness was assessed using Spitting test. Plaque Index was used for evaluating patient`s oral hygiene. Examinations were performed on a dental unit using WHO-approved equipment and methods. In case of acute, severe and life-threatening complications, the patient was referred to an appropriate care center. The frequencies of dental and oral problems including buccal petechia, pharyngeal erythema/hyperplasia, hairy tongue, herpes, candidiasis, aphthous ulcers, oral habits, reflux, mouth dryness, and oral irritation, malodor and bleeding were determined in both groups and compared. Subjects with oral complications were referred to the respective specialist physician for further assessment, differential diagnosis, and treatment. Possible association between oral lesions and drugs used in SM-exposed patients was also evaluated.

The study protocol was reviewed and approved by the institutional Ethics Committee at the Baqiyatallah University of Medical Sciences, Tehran, Iran.

Data were analyzed using SPSS software, version16 (SPSS Inc., Chicago, IL, USA). Between-group comparisons were made using Chi-square test for categorical variables, and independent samples t-test for numerical variables.

## Results

All studied subjects were male. The mean age in the total study population was 44.97±4.45 years (age range: 36-64 years). Mean age in case and control groups were 44.58±4.5 years, and 45.38±4.38 years, respectively. The groups were comparable with respect to age. Out of the total study population, 100 patients had pulmonary complications, 75 skin symptoms, 48 ocular complications, 32 neurologic symptoms, and 12 had other symptoms. Mean percentage of disability was 45.75±15.51% in case group. In the case group, 10 subjects were smoker compared with 22 subjects in the control group, being significantly different between the groups (*p*<0.05). Thirty-nine patients in case group and 34 in control group did not have high school graduation, which was comparable between the groups (*p*>0.05). Seventy-three patients in case group and 71 in control group had dental visits in the preceding year, which was not significantly different between the groups (*p*>0.05). Overall, there was no significant difference between the case and control groups regarding oral hygiene status according to the WHO Oral Health Assessment Criteria (*p*>0.05) ( Table 1). The frequencies of buccal petechia, pharyngeal erythema/hyperplasia, hairy tongue, candidiasis and reflux disease were considerably higher in the case vs. control group (*p*<0.05). However, case and control groups were comparable with respect to the frequencies of herpes infection, aphthous ulcers, mouth dryness, oral irritation, oral malodor, and oral bleeding (*p*>0.05). The percentage of disability was significantly higher in the subgroup of subjects with candidiasis compared with candidiasis-free subgroup (*p*<0.05). Prevalence of hairy tongue and pharyngeal erythema/hyperplasia were higher in the subgroups of patients taking antibiotics and salmeterol, respectively (*p*<0.05). The mean numbers of lost teeth (5.67±4.69 in case group and 5.29±3.94 in control group) and filled teeth (6.28±3.22 in case group and 5.76±4.28 in control group) were not significantly different between the groups (*p*>0.05) ( Table 2). The prevalence of antibiotic and corticosteroid use in the case group was 27% and 58%, respectively.

**Table 1:**
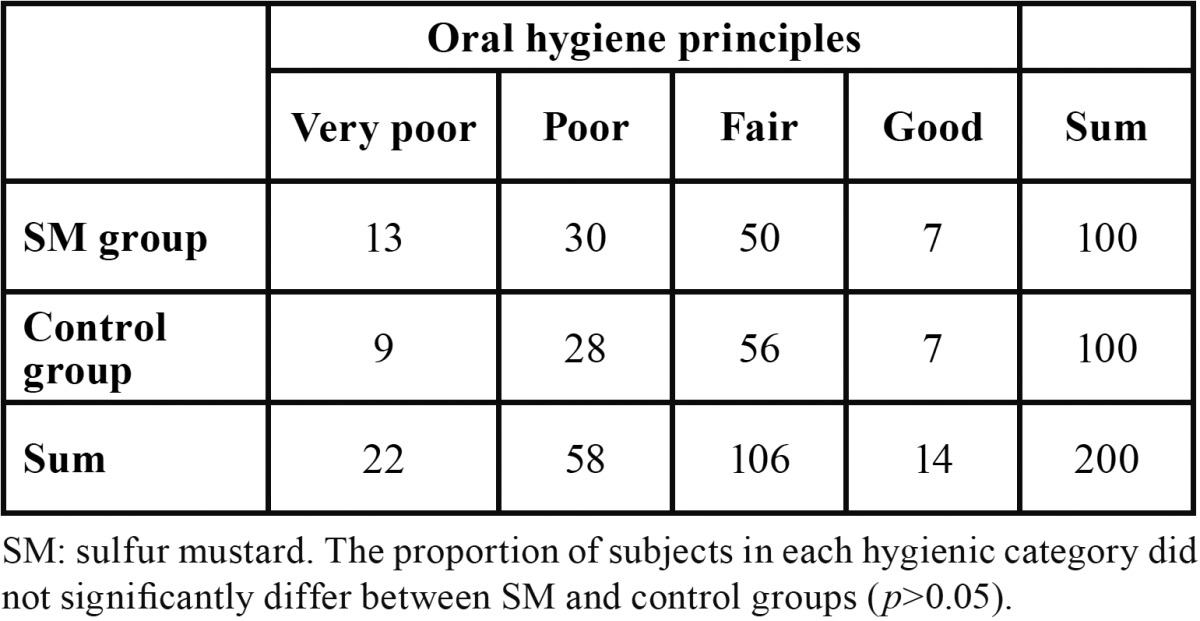
Distribution of patients based on oral hygiene principles.

**Table 2:**
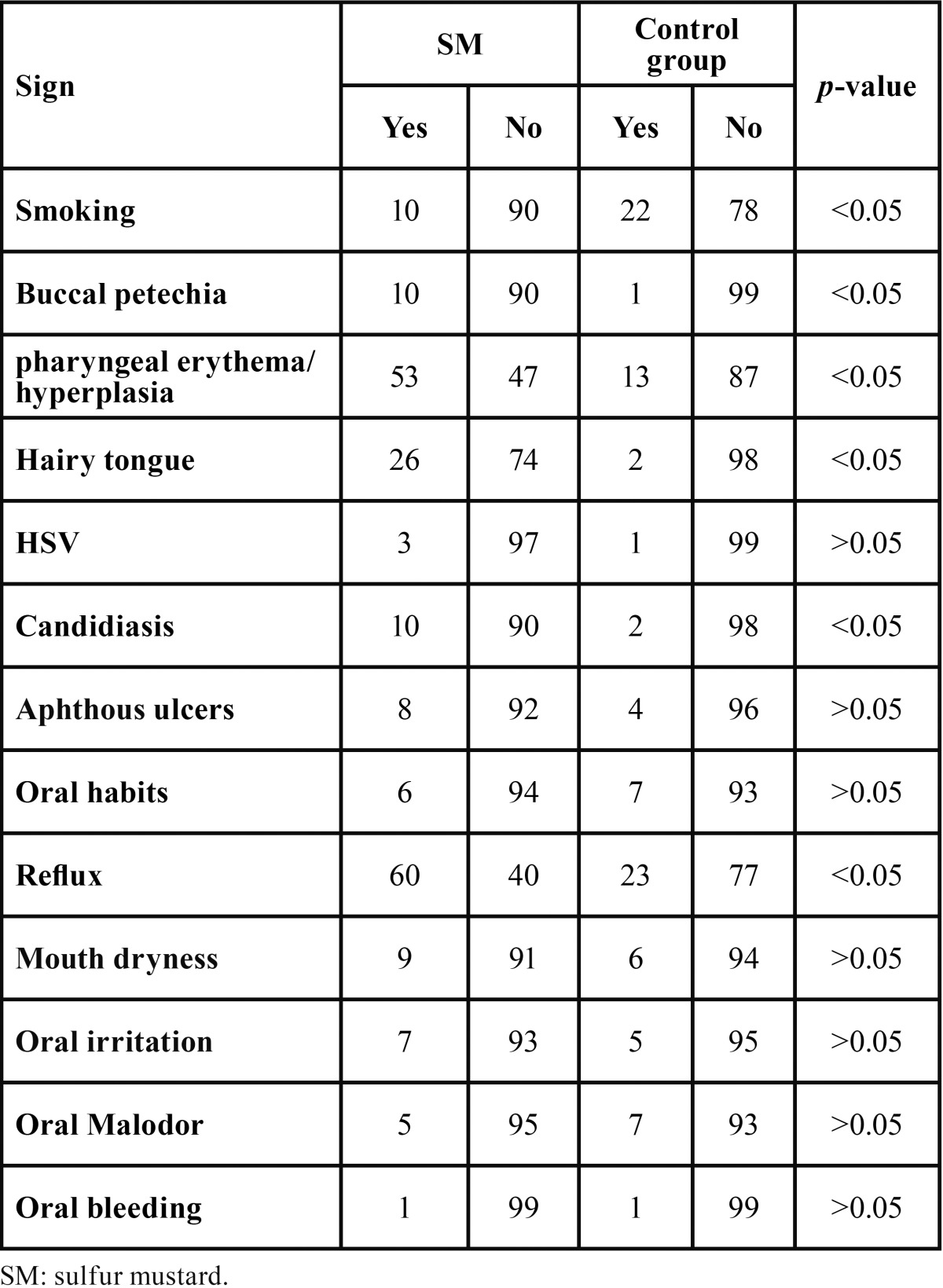
Frequency of oral abnormalities in the study groups.

## Discussion

In order to fulfill the objective of the present study, oral hygiene status of patients exposed to SM was compared with age-matched non-exposed individuals. All SM-exposed patients had pulmonary symptoms which is indicative of the sensitivity of lungs to toxic effects of SM. In addition to respiratory problems, dermal, ocular and neurological complications were also observed. Patients exposed to SM smoked significantly less than the control group, which may itself be a consequence of numerous pulmonary problems causing inability of subjects to smoke. The association between cigarette smoking and several oral problems such as periodontitis, hairy tongue, leukoplakia, has been shown in previous studies (15,16). Smoking may potentially serve as a confounding factor for the higher frequencies of some of the symptoms in the case group. However, it is not likely that the higher prevalence of symptoms such as candidiasis in the case group that was observed in the present study is related to smoking because the number of smokers was significantly fewer in case vs. control group. The exact etiology for the higher rate of buccal petechia and pharyngeal erythema and/or hyperplasia in the SM-exposed group remains to be investigated by future studies. It is known that SM can cause hematological disturbances in both acute and chronic phases. In a large cohort among SM-exposed civilians of Iran, a significantly reduced platelet count was reported 20 years after exposure, however that thrombocytopenia is a constant chronic feature of SM exposure has not been a consistent finding (17,18). Hairy tongue lesions were more prevalent in SM exposed patients; most probably as a result of higher antibiotic use in these patients. The association between extensive use of antibiotics and hairy tongue lesions has been previously demonstrated (9). Higher prevalence of candidiasis in the case group is probably due to immune suppression and simultaneous use of antibiotics and corticosteroids. There was only one case of squamous cell carcinoma located in the posterior part of the tongue in the case group, with known etiology (constant stimulus with a broken tooth), and had no relation to SM exposure or consumed drugs. Prescribed drugs in the case group were salbutamol, salmeterol, ipratropium bromide, fluticasone, beclomethasone and other corticosteroids, antibiotics, psychiatric drugs, methylxhantines and omeprazole. The rate of adherence to oral hygiene principles was comparable between the groups. This finding may be due to the proper dental care facilities granted to patients exposed to SM gas by the Veteran’s Affair of Iran (Janbazan Foundation). In the present study, the percentage of disability was only related to oral candidiasis but not other symptoms such as petechial lesions, pharyngeal erythema and herpes. Patients with higher percentages of disability had a greater risk of candidiasis infection, most probably because of their depressed cell-mediated immunity as reported previously (19-22). This immune suppression is the consequence of both SM’s direct effects and the impact of immunosuppressive medications used by patients (21). With respect to the direct effects of SM, several immunological imbalances have been reported in subjects suffering from long-term complications of SM, including increased titres of C3, C4, IgG and IgM, and depressed cell-mediated immunity characterized by reduced number of natural killer cells (19-22).

Taken together, SM exposure was associated with increased prevalence of oral candidiasis. However, this complication along with others due to the side effects of prescribed medications (pharyngeal erythema/hyperplasia and hairy tongue) were not found to cause long-term dental decays, dental loss, or increased intra/extra oral lesions. The observed association between the use of salmeterol and pharyngeal erythema/hyperplasia may necessitate drug switching to other bronchodilators in SM-exposed subjects, or reducing salmeterol’s dose. Finally, in patients with higher percentages of disability, care should be taken to prevent and treat oral candidiasis with antifungal drugs such as nystatin. Owing to the multi-organ damage and long-term complications of SM, continuous follow-up of exposed subjects is necessary. Adherence to oral health principles and routine examinations by dentist is vital to prevent the development of oral lesions and dental caries.
